# Identifying the determinants of premature mortality in Russia: overcoming a methodological challenge

**DOI:** 10.1186/1471-2458-7-343

**Published:** 2007-11-28

**Authors:** Susannah Tomkins, Vladimir Shkolnikov, Evgueni Andreev, Nikolay Kiryanov, David A Leon, Martin McKee, Lyudmila Saburova

**Affiliations:** 1 Department of Epidemiology and Population Health, London School of Hygiene and Tropical Medicine, London; 2 Max Plank Institute for Demographic Research, Rostock, Germany; 3 Izhevsk Medical Academy, Izhevsk, Russia; 4 Department of Public Health and Policy, London School of Hygiene and Tropical Medicine, London; 5 Social Technologies Institute, Izhevsk, Russia

## Abstract

**Background:**

It is thought that excessive alcohol consumption is related to the high mortality among working age men in Russia. Moreover it has been suggested that alcohol is a key proximate driver of the very sharp fluctuations in mortality seen in this group since the mid-1980s. Designing an individual-level study suitable to address the potential acute effects of alcohol consumption on mortality in Russia has posed a challenge to epidemiologists, especially because of the need to identify factors that could underlie the rapid changes up and down in mortality rates that have been such a distinctive feature of the Russian mortality crisis. In order to address this study question which focuses on exposures acting shortly before sudden death, a cohort would be unfeasibly large and would suffer from recruitment bias.

**Methods:**

Although the situation in Russia is unusual, with a very high death rate characterised by many sudden and apparently unexpected deaths in young men, the methodological problem is common to research on any cause of death where many deaths are sudden.

**Results:**

We describe the development of an innovative approach that has overcome some of these challenges: a case-control study employing proxy informants and external data sources to collect information about proximate determinants of mortality.

**Conclusion:**

This offers a set of principles that can be adopted by epidemiologists studying sudden and unexpected deaths in other settings.

## Background

It is thought that excessive alcohol consumption is related to the high mortality among working age men in Russia. Specifically, it has been suggested that alcohol is a key proximate driver of the very sharp fluctuations in mortality seen in this group since the mid-1980s including a seven year period when, on average, male life expectancy at birth fell by more than one year for each calendar year [1-4]. Most of the evidence comes from the analysis of routine data and from cross-sectional surveys of alcohol consumption, while in contrast there have been relatively few studies linking the drinking behaviour of individuals to mortality in Russia. Extrapolating the alcohol-mortality relationships found in research undertaken in Western countries to Russia is likely to be inappropriate because, along with many other parts of the former Soviet Union, it has a particular pattern of drinking characterised by binge-drinking of large volumes of spirits on one occasion, a pattern that is believed to be particularly dangerous [5].

## The challenge

Designing an individual-level study to investigate these issues raises many methodological challenges. The initial hypothesis is that the sharp fluctuations in working-age mortality that have characterised Russian demographic trends since the mid-1980s may be due to acute or very short-term effects of alcohol consumption. An individual's pattern of drinking may also change considerably over a short period of time. Thus, what is required is a design for collecting valid information about individual characteristics and drinking behaviours that can then be related to the risk of death in the following 12 or 18 months.

The two classic analytic study designs are cohort and case-control studies. However, in their conventional forms, neither design is ideal to address this problem. Cohort studies typically measure exposure at a base-line examination or interview and then follow up the study subjects. If mortality is the endpoint, follow-up is usually for years. This design is particularly suited to studying mortality effects of relatively stable patterns of exposure, such as smoking, or acute or episodic exposures which elevate mortality over an appreciable period of time such as exposure to radiation and subsequent risk of solid tumours. In the case of short-term mortality effects of acute and intrinsically unstable patterns of behaviour including binge drinking, a conventional cohort design is not optimal. As follow-up time accumulates, classification of people according to baseline reports of frequency and intensity of binge drinking becomes increasingly uninformative about exposures that may be occurring shortly before death. One possible solution to this would be to have a cohort study of such size that sufficient numbers of events would accrue within a short period of follow-up. In most instances the huge size of the required cohort would make this unfeasible. Another alternative would be to undertake regular resurveys of the cohort to update exposure information about drinking patterns, although this would be logistically difficult, expensive and likely to result in selective loss to follow-up of heavy drinkers as discussed below.

Some cohort studies suffer from another problem in terms of their suitability for studying the mortality effects of heavy drinking, or other outcomes associated with behavioural problems and social dysfunction. This is the selective exclusion of those at the ends of the spectrum, such as heavy or problem drinkers [6]. In a study which relied on regular resurveys, those individuals who became heavier/more problematic drinkers since being captured at baseline would be less likely to take part in resurveys than others. Additionally, studies for which recruitment requires subjects to travel from their home address, to an appointment in a clinic for examination for example, are also likely to exclude problem drinkers differentially. This is a well recognised phenomenon in cross-sectional studies, but may be particularly serious when cohort recruitment requires considerable commitment from the potential participants. In the Russian context, two of the larger and well known cohorts (the Lipid Research Clinics and the Novosibirsk cohorts) [7,8] both required subjects to attend for a medical examination as part of the recruitment protocol. These studies are likely to have differentially excluded people who have serious alcohol problems at the time of recruitment.

In contrast, a case-control study in this context would focus upon obtaining information on the behaviours and characteristics of subjects in the most recent 12 month period, which for the cases (deaths) would be the year before death. From this perspective, the case-control design is more suitable than a cohort design. Certainly, retrospectively collected information about the immediate past might be regarded as more reliable and accurate than information about behaviours and characteristics much further back in time, as is often attempted in case-control studies.

If a case-control design is chosen it will require a departure from the usual approaches as, obviously, it must use proxy informants. Examples of such studies exist in the literature, particularly in investigations of risk factors for suicide [9,10] and violent death [11,12]. Other risk factors including drug use, smoking [13-15]and dietary factors [16] have also been explored using case-control designs with proxy informants. There is less literature on differential response bias between cases and controls based on proxy interviews compared to when the index cases and controls are themselves interviewed [17-19], and it is almost impossible to evaluate the extent of such bias when cases have died.

A case-control study in which the cases are dead does, however, have an advantage compared to the majority of studies where cases are of diagnosed disease. In a population with a well-functioning vital registration system, 100% case-ascertainment is feasible. Moreover even if detailed proxy-based information is not available for all, there will usually be sufficient routine data collected at death registration to assess the representativeness of the cases with full information. If the target group of cases comprises all deaths occurring in a defined population, identifying the appropriate control sampling frame is straightforward: it is the whole population of the area in which the deaths are from.

Our interest in alcohol as the main exposure presents problems for case-control studies as it does for cohorts, particularly if, as is the case here, we wish to look at the role of heavy drinking. As already mentioned above cross-sectional surveys are likely to fail to interview people who drink heavily differentially. In terms of actual methods, a case-control study is very similar to a cross-sectional study in that one approaches people to be interviewed without a history of prior contact and engagement in the research. However, the obligatory use of proxies may actually help in one respect – in that the drinking behaviours of the cases and controls may be less strongly linked to the process of recruitment of proxies than they are to recruitment of the index subjects themselves.

In the rest of this paper we describe the design and implementation of an innovative study in Russia that illustrates how these challenges and issues can be addressed. Although some aspects of the design reflect the specific situation in Russia, we believe that it offers a set of principles that can usefully be adopted by epidemiologists studying sudden and unexpected deaths in other settings.

## The Izhevsk Family Case-control study

The Izhevsk Family Study was established to investigate whether patterns of alcohol consumption were linked to short-term risk of death among Russian men of working age in a typical, medium-sized Russian industrial town, Izhevsk. As already highlighted, the key challenge was to design a study which was able to capture information about exposures hypothesised to lead to mortality in the period immediately prior to death and to obtain a representative set of living controls with which to compare them.

Izhevsk has a population of 650 000 people and is located on the western side of the Ural mountains. It is the capital of the Udmurt Republic, one of the 89 territories that make up the Russian Federation. The leading causes of death among these men were cardiovascular disease, a high proportion of which were sudden, and external causes. While the focus was on alcohol, clearly it was important to be able to collect information on other factors that might act at different points on the causal pathway, such as unemployment, or act as confounders, such as smoking.

The Izhevsk Family study is not the first study to address the issue of premature mortality among Russian men of working age. The study benefited from an earlier study of 1998–99, also based in the Udmurt Republic, that attempted to address the same issue [4,20]. The previous study used broadly the same design, and found an association between excessive alcohol consumption and premature mortality (below age 55 years) in Russian men. However, this previous study had several weaknesses which threw these findings into question. For example, despite including several questions about alcohol use, the information was limited and inadequate; it included too many subjective/evaluative questions, to which proxies could not reliably respond; no checks were made regarding whether the cases belonged to the sampling frame for the controls which could have causes selection bias; no data was collected from external, objective sources, nor were the controls themselves interviewed, so there was no opportunity to validate proxy-obtained data. In comparison, the Izhevsk Family study was strengthened in a number of important respects. Firstly, the questionnaire was improved, excluded subjective/evaluative questions and incorporated more questions on clearly observable behaviours, so as to obtain more valid responses from proxy respondents. Secondly, the questionnaire included an extended range of questions on alcohol consumption and alcohol-related behaviour, including questions about surrogates (manufactured alcohol-containing substances not intended for drinking), the consumption of which subsequently emerged as particularly important. Thirdly, the Izhevsk Family study carried out interviews with both controls and their proxies, and in addition previously collected administrative and clinical data on the subjects was obtained, providing opportunity for validation of responses obtained from proxy respondents. Finally, it was possible to identify which cases belonged to the sampling frame from which controls were drawn, and hence sensitivity analyses could be conducted.

### Study design

Given the above considerations, a population-based case control design was chosen. Ethical approval for the study was obtained from the committees of the Izhevsk Medical Academy and the London School of Hygiene & Tropical Medicine. Identification of deaths from all causes (cases) occurring over a two-year period (2003–5) was straightforward, as all deaths were registered with the city vital registration bureau (ZAGS). Controls could be selected from computerised voters lists and as these include not only name, address, and sex, but also date of birth, it was possible to frequency-match to the cases by age group. A total of 1750 case proxies and 1750 control proxies were recruited and interviewed. This represented an overall response rate of 62% among cases (1750/2835 total identified deaths), and 57% among controls (1750/3078 control household approached). Detailed information on recruitment and response rates has been previously reported [21]. Table 1, below, shows the distribution of cases and controls by age group.

**Table 1:** Age distribution of subjects

Age group	Case		Control	
	N	%	N	%

25–29	131	7.49	132	7.54
30–34	144	8.23	145	8.29
35–39	137	7.83	140	8
40–44	305	17.43	294	16.8
45–49	441	25.2	434	24.8
50–54	592	33.83	605	34.57

Total	1,750	100	1,750	100	5,191	100

### Obtaining information on subjects

Since cases were men who had died, information about them was obtained by interviewing proxy informants. Proxies were also interviewed for all controls, in order to use the same method of data collection. The use of proxies, however, raises issues of the validity of the information they provide about the index subject. At the design stage this was addressed by means of a systematic review of studies that had looked at the extent of agreement between subjects and proxies [22]. The review concluded that, for most of the factors in which we were interested and in particular, alcohol and tobacco consumption, there was evidence from the literature that levels of agreement would be acceptable provided questions were carefully constructed to focus upon index behaviours that the proxy would be able to directly observe. Therefore, in addition to conventional quantity-frequency questions about alcohol consumption, we also developed and used questions which acted as markers of hazardous alcohol consumption, including behaviours such as the frequency with which the subject had fallen asleep with his clothes on because they were drunk, and how often the subject had had a hangover or had been excessively drunk.

The review also indicated that those closest to the index case, typically spouses, tended to provide the most reliable and valid information about the index subject. Thus, selection of controls involved identification of the 'best informant', defined as someone who had been living with the index for unbroken period of at least 6 months at the current time/at the time of death (for cases). Where there was more than one potential informant with comparable duration of co-residence, the individual was selected according to their relationship to the index, in the order prescribed by the findings of the systematic review: wife/girlfriend/partner, sister, mother, brother, father, offspring, other, as shown in Table 2. The questionnaire included a section about the proxy's own socio-demographic characteristics, since the choice of proxy can influence the validity and reliability of data obtained. Where the index lived alone they were excluded by necessity, which affects the generalisability of the results. If, however, the index had recently moved out of his permanent residence to a new address without the rest of his household or the potential informants had recently moved away from, or in to, the index's permanent residence and had therefore not lived with the index continuously for the previous 6 months, interviewers followed a series of detailed steps to ensure a consistent proxy selection process. In only 15 case households and 4 control households, interviews were carried out with a proxy who had not lived with the index for an unbroken period of 6 months, illustrating that this protocol was successfully followed by the fieldworkers.

**Table 2:** Relationship of proxy to index

**Relationship to index**	**Case**		**Control**		**Total**	
	N	%	N	%	N	%

Wife/girlfriend/partner	1,036	59.2	1,486	84.9	2,522	72.1
Mother	328	18.7	152	8.7	480	27.4
Father	27	15.2	9	15.3	36	30.4
Sister	68	3.9	14	0.8	82	2.3
Brother	51	2.9	10	0.6	61	1.7
Daughter	79	4.5	23	1.3	102	2.9
Son	55	3.1	35	2.0	90	2.6
Daughter/son in law	3	0.2	1	0.1	4	0.1
Other	103	5.9	20	1.1	123	3.5

Total	1750	100	1750	100	3500	100

The distribution of proxies for cases and controls differs, whereby more spouses were available for controls, whilst mothers tended to be the best available proxies for cases. This reflects the inherent difference in marital status and household composition between these two groups. Whilst it is possible that this introduced some bias into the sample due to the possible differences in reporting by different proxy types, provided the proxy fulfils certain criteria related to their ability to reliably report on the index, this will be minimal [22]. Analyses restricted to spouses only obtained very similar results to those which were unrestricted according to proxy type [21]

### External validation

In our study, given its focus on hazardous drinking and its potential antecedents, it was desirable to obtain independent information on this issue. This was considered to be particularly relevant to assessing a) differential reporting by proxies by case-control status; b) representativeness of the cases and controls for whom proxy interviews were obtained. Direct evidence of a history of alcohol abuse was obtained from records of treatment in the city Narcology Dispensary (the alcohol and drug treatment clinic). In addition, external information was obtained from two other sources: evidence of any prison stays was obtained from police records; evidence of current benefits obtained and disability status were collected from the Social Security bureau. These made it possible to go well beyond the usual socio-demographic characteristics used when testing for recall bias. Our use of external data is illustrated by the example in Table 3 which shows the association between registration at the Narcology Dispensary and one indicator of hazardous drinking, consumption of surrogates, among cases and controls. Use of external data also provided an opportunity to fill an important gap in the literature on proxy informants by asking not just whether indexes and proxies agreed but also which were more accurate and, exceptionally, doing so in relation to issues that are often considered stigmatising and therefore subject to biased informant response [22].

**Table 3:** Association between Narcology Dispensary registration and surrogate consumption among cases and controls

	Cases narcology registration		Controls narcology registration	
Surrogate consumption	No	Yes	No	Yes
No	904	84	1,549	41
Yes	518	199	110	25
Don't know/difficult to answer	36	9	20	5
Chi-square*	111.14		97.78	
p value	<0.001		<0.001	

*chi-squared test for a general association between reported surrogate consumption and registration at the Narcology Dispensary

All three sources of data are administrative, so are not prone to the subjective biases in questionnaire responses. Since information was recorded prior to death, it could not have been affected by the fact of death itself. Further information on these sources of data is detailed in Table 4.

**Table 4:** external sources of data

**Narcology Dispensary.**
• Registration at the Izhevsk Narcology Dispensary results in an official record of registration. • Circumstances leading to registration include being arrested for an alcohol-related offence, compulsory referral by a doctor at a polyclinic or hospital, or voluntary registration by self, or the family of a patient. • Many of those who display hazardous drinking behaviours are registered for treatment at the Narcology service at some point.
**Social Security bureau**
• This bureau holds official records of any disability benefit of which people are in receipt. • The list is definitive and exhaustive, since it is the means by which payments are regulated. • Since the purpose of this list is to control financial payments to citizens, it is believed to be accurate and complete. • Records are identifiable by name, date of birth and address, allowing reliable record linkage. • Details include class of disability and date of registration. • These data can be compared with the questionnaire item on disability registration.
**The City of Izhevsk Police database**
• This database holds all records of prison stays. • It is unaffected by bias, since there are no means by which a record can be avoided if a person has spent time in prison. • Again, records are identifiable by name, date of birth and address, allowing reliable record linkage. • Presence/absence of a record can be compared with the questionnaire item regarding whether the index has ever spent time in any prison.

### Information bias

Problems with validity of reporting behaviours are well documented, especially when the respondents are proxies, and the behaviours of interest are culturally sensitive, as alcohol consumption may be considered to be. As described above, steps were taken to quantify the extent of any information bias by comparing questionnaire responses with external sources of information related to reported behaviours, such as registration at the Narcology Dispensary and alcohol consumption. Given that information on cases was obtained from proxies, it was important, for consistency, that proxies were also used to elicit information on controls. However, since the controls themselves could also be interviewed, it was possible to extend the existing literature on proxy informants by conducting interviews with them and comparing their own responses to those of their proxies [22]. This is illustrated by Table 5 which shows the index-proxy agreement for frequency of consuming spirits in a sample of 1564 control households for which complete information was available. In addition, interviews conducted with an additional proxy in a sample of 200 case and 200 control households were a particularly valuable resource in terms of being able to investigate whether it was characteristics of the proxy, or characteristics of the household in which the index lived which affected the response obtained from the proxy. This is illustrated by Table 6, which shows the high proxy-proxy agreement for a question on current smoking status of the index in a sample of 131 case and 178 control households.

**Table 5:** Agreement between control indexes and proxies about frequency of surrogate consumption among 1564 control households with complete information for both respondents

	Control							
Control proxy	Every day or more often	Nearly every day	3–4 times per week	Once or twice a week	1–3 times per month	A few times per year	Never or almost never	Total

Every day or more often	2	0	1	2	1	1	0	7
Nearly every day	1	11	8	12	8	4	0	44
Three or four times a week	0	2	14	39	15	3	3	76
Once or twice a week	0	5	17	159	103	15	3	302
1–3 times a month	1	4	7	100	284	66	12	474
A few times per year	1	4	0	22	100	187	21	335
Never or almost never	0	3	1	8	13	32	269	326

Total	5	29	48	342	524	308	308	1,564

Weighted Cohen's kappa coefficient for agreement between 1564 index-proxy pairs = 0.61

**Table 6:** Agreement between proxy 1 and proxy 2 on current smoking status case and control households

	Case households				Control households			
	Proxy 1		Proxy 2		Proxy 1		Proxy 2	

	N	%	N	%	N	%	N	%

Never a smoker	15	11.5%	16	12.2%	35	19.7%	38	21.3%
No, ex-smoker	9	6.9%	10	7.6%	31	17.4%	27	15.2%
Yes, a current-smoker	107	81.7%	105	80.2%	112	62.9%	112	62.9%
Difficult to answer	0	0.0%	0	0.0%	0	0.0%	1	0.6%
Refuse to answer	0	0.0%	0	0.0%	0	0.0%	0	0.0%
No answer	0	0.0%	0	0.0%	0	0.0%	0	0.0%
*Cohen's kappa coefficient*	*0.96*				*0.87*			

### Selection bias

A challenge every case-control study must overcome is ensuring that the cases and controls are drawn from the same underlying population. Since these two groups are often recruited through different routes, it can be difficult to be certain that case-control selection bias is not affecting analyses. In order to address this issue, within the Izhevsk Family Study, analyses were repeated with a restricted sample which comprised only the cases (who had been originally identified via ZAGS) successfully located within the electoral register – the sampling frame used to select controls.

**Figure 1:**
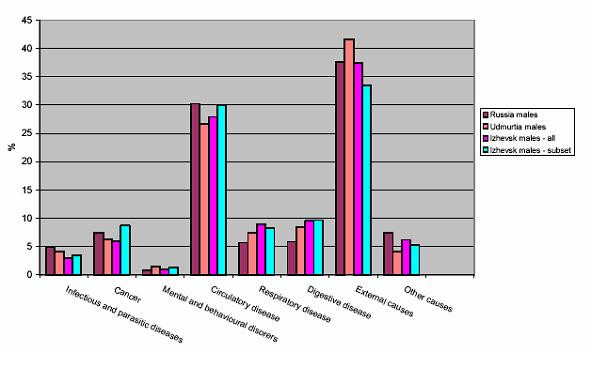
Distribution of deaths by ICD10 chapter among the sample analysed, men living in Izhevsk, men living in Udmurtia and men living in Russia aged 25–54.

It must be noted that whilst we have argued that a case-control approach is more appropriate to answer a study question such as this than a cohort design, the difficulties of such research also render this far from ideal. This is illustrated by the difficulties encountered in recruiting cases. Of a total of 2837 deaths in men aged 25–54 in Izhevsk in the period during which this research was carried out that were eligible for inclusion, almost 15% (415) lived alone and could not be included in this study, 10% (284) met with respondent refusal, and the correct address could not be identified for 13% (378). This means that our analyses were finally based on around 62% of eligible deaths, and there are likely to be some specific biases present, although the same percentage of cases and controls not interviewed were registered at the Narcology Dispensary [21]. Despite this, the profile of mortality by cause in the sample analysed is similar to that for all men living in Izhevsk, all men living in the Udmurt Republic, and all men living in Russia in the age group of interest, as illustrated by Figure 1.

### Additional information on case characteristics

While first level analyses focused on deaths from all causes, it was clearly important to be able to look at specific causes of death as entered on the death certificate by the certifying doctor, or where an autopsy was conducted, from the outcome of this. In Russia all deaths are registered and, most of deaths in the age group of interest (25–54) undergo autopsy, with most being forensic. Cause of death is coded using the 10^th ^revision of the International Classification of Diseases. Yet death certificates provide only limited information and may not include information on pathological features of interest, such as alcoholic liver disease, where this was not directly associated with death. Hence, a structured proforma was designed to collect detailed data obtained at autopsy, including blood alcohol levels. This and other data regarding the circumstances of death provided an opportunity for additional population-based analyses concerning the problem of premature mortality in Russia, including analysis of blood-alcohol concentration at time of death or specific cause of death, and enabled assessment of the validity of the certified cause of death. Whilst some such analyses are separate from the main case-control analysis, they enrich the data, providing important additional information and the prospect of validation of questionnaire-obtained data

## Conclusion

To address the methodological challenge outlined in this paper, we adopted a population-based case-control design. We started with all deaths in men aged 25–54 (and a representative sample of live controls) from an entire defined population. We were therefore able to assess whether or not the cases and controls for whom we could not obtain an interview were representative of the target population. A great strength of this study was therefore that it was not subject to the same selection biases typically encountered by this type of research.

Although based on a conventional case control study, this study has three unusual features. First, although information could not be obtained from cases, it could be obtained from proxy informants providing certain precautions were taken. It was important to select proxies who had sufficient knowledge of the index to be able to report on their characteristics and behaviours, and to pose questions that addressed characteristics and behaviours that were observable. Second, given the need to ensure representativeness of controls and tackle recall bias, external sources of objective data relevant to the exposures being studied but blind to case/control status were used. Third, to overcome potential problems in defining outcomes, a detailed system of data collection to validate and extend information on causes of death was employed. Although adapted to the specific circumstances of Russia, we believe that the principles that we have set out here will be helpful to others seeking a better understanding of the causes of conditions associated with sudden and unexpected death in a range of situations, including deaths at young and middle ages, or deaths from certain causes such as violence or accidents, or tuberculosis. Moreover, the study illustrates that with sufficient attention given to issues of data quality and bias at the design stage, ancillary information can be collected which can be used to assess and demonstrate the validity of data collected using methods that might otherwise be regarded as questionable.

## Competing interests

The author(s) declare that they have no competing interests.

## Authors' contributions

The idea of the study was developed by VS with input from DL and MM. All authors contributed to the detailed design of the protocol. LS was responsible for the conduct of the interview field work, NK for coordination of all other data collection in Izhevsk, EA for data capture systems and monitoring of field work progress, ST for the detailed design of the questionnaire (including the systematic review of proxy validity) and coordination of the project and management of the final data set and MM for the autopsy and setting up of the surrogate toxicological analyses. ST drafted the text which was commented upon by all authors. All authors read and approved the final manuscript.

## Pre-publication history

The pre-publication history for this paper can be accessed here:



## References

[B1] Chenet L, McKee M, Leon D, Shkolnikov V, Vassin S (1998). Alcohol and cardiovascular mortality in Moscow; new evidence of a causal association. J Epidemiol Community Health.

[B2] Leon DA, Chenet L, Shkolnikov VM, Zakharov S, Shapiro J, Rakhamova G, Vassin S, McKee M (1997). Huge variation in russian mortality rates 1984-94: artefact, alcohol, or what?. Lancet.

[B3] McKee M, Shkolnikov VM, Leon DA (2001). Alcohol is implicated in the fluctuations in cardiovascular disease in Russia since the 1980s. Annals of Epidemiology.

[B4] Shkolnikov V, McKee M, Chervyakov VV, Kyrianov NA (2002). Is the link between alcohol and cardiovascular death among young Russian men attributable to misclassification of acute alcohol intoxication? Evidence from the city of Izhevsk. Journal of Epidemiology and community health.

[B5] Rehm J, Taylor B, Patra J (2006). Volume of alcohol consumption, patterns of drinking and burden of disease in the European region 2002. Addiction.

[B6] Gmel G, Rehm J (2004). Measuring alcohol consumption. Contemporary Drug Problems.

[B7] Dennis H, Zhukovsky GS, Shestov DB, Davis CE (1993). The association of Education with Coronary Heart Disease Mortality in the USSR Lipid Research Clinics Study. International Journal of Epidemiology.

[B8] Malyutina S, Bobak M, Kurilovitch S, Ryizova E, Nikitin Y, Marmot MG (2001). Alcohol consumption and binge drinking in Novosibirsk, Russia, 1985-95. Addiction.

[B9] Dumais A, Lesage AD, Alda M, Rouleau G, Dumont M, Chawky N, Roy M, Mann JJ, Benkelfat C, Turecki G (2005). Risk factors for suicide completion in major depression: a case-control study of impulsive and aggressive behaviors in men. Am J Psychiatry.

[B10] Kung HC, Pearson JL, Liu X (2003). Risk factors for male and female suicide decedents ages 15-64 in the United States. Results from the 1993 National Mortality Followback Survey. Soc Psychiatry Psychiatr Epidemiol.

[B11] Rivara FP, Mueller BA, Somes G, Mendoza CT, Rushforth NB, Kellermann AL (1997). Alcohol and illicit drug abuse and the risk of violent death in the home. Journal of the American Medical Association.

[B12] Rowe B, Milner R, Johnson C, Bota G (1994). The association of alcohol and night driving with fatal snowmobile trauma: a case-control study. Ann Emerg Med.

[B13] Chen ZM, Liu BQ, Boreham J, Wu YP, Chen JS, Peto R (2003). Smoking and liver cancer in China: case-control comparison of 36,000 liver cancer deaths vs. 17,000 cirrhosis deaths. Int J Cancer.

[B14] Brennan P, Crispo A, Zaridze D, Szeszenia-Dabrowska N, Rudnai P, Lissowska J, Fabianova E, Mates D, Bencko V, Foretova L, Janout V, Fletcher T, Boffetta P (2006). High cumulative risk of lung cancer death among smokers and nonsmokers in Central and Eastern Europe. Am J Epidemiol.

[B15] Lam TH, Ho SY, Hedley AJ, Mak KH, Peto R (2001). Mortality and smoking in Hong Kong: case-control study of all adult deaths in 1998. British Medical Journal.

[B16] Ho SY, Schooling M, Hui LL, McGhee SM, Mak KH, Lam TH (2006). Soy consumption and mortality in Hong Kong: proxy-reported case-control study of all older adult deaths in 1998. Prev Med.

[B17] Marshall J, Priore R, Haughey B, Rzepka T, Graham S (1980). Spouse-subject interviews and the reliability of diet studies. American Journal of Epidemiology.

[B18] Halabi S, Zurayk H, Awaida R, Darwish M, Saab B (1992). Reliability and validity of self and proxy reporting of morbidity data: a case study from Beirut, Lebanon. International Journal of Epidemiology.

[B19] Graham P, Jackson R (1993). Primary versus proxy respondents: comparability of questionnaire data on alcohol consumption. American Journal of Epidemiology.

[B20] Shkolnikov V, Chervyakov V, McKee M, Leon DA (2004). Russian mortality beyond vital statistics: Effects of social status and behaviours on deaths from circulatory disease and external causes - a case-ctontrol study of men aged 20-55 years in Udmurtia, 1998-99. Demographic Research.

[B21] Leon DA, Saburova L, Tomkins S, Andreev E, Kiryanov N, McKee M, Shkolnikov VM (2007). Hazardous alcohol drinking and premature mortality in Russia: a population based case-control study. Lancet.

[B22] Tomkins S (2006). Proxy respondents in a case-control study: validity, reliability and impact. PhD..

